# Miller Fisher syndrome with bilateral vocal cord paralysis: a case report

**DOI:** 10.1186/s13256-020-2357-4

**Published:** 2020-02-18

**Authors:** Karan N. Ramakrishna, Vikrant Tambe, Adithya Kattamanchi, Amit S. Dhamoon

**Affiliations:** Department of Medicine, State University of New York (SUNY) Upstate Medical University, 750 East Adams Street, Room 5138, Syracuse, NY 13210 USA

**Keywords:** Autoimmune demyelinating polyneuropathy, Acute flaccid paralysis, Vocal cord palsy

## Abstract

**Background:**

Miller Fisher syndrome is a variant of acute inflammatory demyelinating polyneuropathy classically characterized by ataxia, ophthalmoplegia, and areflexia. Miller Fisher syndrome can present with uncommon symptoms such as bulbar, facial, and somatic muscle palsies and micturition disturbance.

**Case presentation:**

We describe the case of a 76-year-old white man with new-onset ataxia, stridor, areflexia, and upper and lower extremity weakness who required intubation at presentation. An initial work-up including imaging studies and serum tests was inconclusive. Eventually, neurophysiological testing and cerebrospinal fluid analysis suggested a diagnosis of Miller Fisher syndrome. Our patient responded to treatment with intravenous immunoglobulin and supportive therapy.

**Conclusion:**

The occurrence of acute or subacute descending paralysis with involvement of bulbar muscles and respiratory failure can often divert clinicians to a diagnosis of neuromuscular junction disorders (such as botulism or myasthenia gravis), vascular causes like stroke, or electrolyte and metabolic abnormalities. Early identification of Miller Fisher syndrome with appropriate testing is essential to prompt treatment and prevention of further, potentially fatal, deterioration.

## Background

Miller Fisher syndrome (MFS) is an uncommon variant of acute inflammatory demyelinating polyneuropathy (AIDP). The classic triad of MFS is ophthalmoplegia, ataxia, and areflexia, which was first described by Miller Fisher in 1956 [1]. It is an important differential diagnosis to consider in patients presenting with acute or subacute flaccid descending paralysis and is sometimes overlooked in favor of other etiologies, such as stroke, myasthenia gravis (MG), electrolyte abnormalities, and botulism. Below, we describe a case of MFS with an atypical presentation of respiratory failure due to vocal cord paralysis, in addition to the classically reported ataxia and areflexia.

This report is an illustration of an unusual bulbar presentation of an uncommon variant of AIDP, being only the second reported instance of vocal cord paralysis due to MFS. The intent of this case report is to encourage clinicians to not discount the possibility of a rare but potentially treatable inflammatory demyelinating disorder when encountering unexplained cranial nerve symptomatology.

## Case presentation

A 76-year-old white man presented to our emergency department (ED) with recent onset of unsteady gait, poor oral intake, dysarthria, and dizziness for 1 week prior to presentation. His wife called an ambulance and had him brought to our ED as she noticed he was short of breath for the prior 12 hours. He did not have any symptoms suggestive of a recent upper respiratory tract infection or gastroenteritis. He denied exposure to sick contacts, recent immunizations, or consumption of canned foods or beverages. He did have a past medical history significant for esophageal stricture (diagnosed 2 years before) for which he had been undergoing routine endoscopic balloon dilation (last attempt was 3 months before). He lived in a rural upstate New York county and had retired as a meat-cutter 15 years before. His only medications at the time were ferrous sulfate (324 mg daily), pantoprazole (40 mg twice daily), and a multivitamin. He did not have a history of tobacco smoking. He consumed approximately two standard drinks in a week. He had no known allergies. While in our ED, his vital signs were: temperature, 37 ºC (98.6 ºF); heart rate, 100–110 beats per minute; blood pressure, 141/82 mmHg; and oxygen saturation 82% on room air and requiring 100% fraction of inspired oxygen (FiO_2_) via facemask to maintain saturations above 96%. On physical examination, he was in marked respiratory distress but not toxic-appearing. He was awake but drowsy, oriented only to self. His pupils were equal and symmetrical with appropriate response to light. His extra-ocular movements were intact. There was no facial droop; there was no deviation of tongue or uvula. His gag reflex was impaired. He could move all four extremities spontaneously and on command. However, motor strength appeared to be symmetrically and mildly diminished in all extremities and accompanied by decreased tone. Diminished knee and ankle reflexes were noted bilaterally. He had a diminished flexor plantar response bilaterally. Sensation was grossly intact in his face, trunk, and extremities. Coordination was impaired in upper and lower extremities with dysmetria and dysdiadochokinesia. Gait was not assessed. He had no visible involuntary movements. His neck was supple without signs of meningismus. A cardiovascular examination was unremarkable, with normal heart sounds and equal symmetrical pulses in bilateral extremities. His abdominal, musculoskeletal, and skin examinations were unremarkable. Although he was hemodynamically stable, he became lethargic with stridor and progressive hypoxia. Given concern for airway protection, he was emergently intubated with a 7.5 mm endotracheal tube on first attempt and placed on mechanical ventilatory support. For intubation, he was administered 1.5 mg/kg of succinylcholine and 0.3 mg/kg of etomidate intravenously. Maintenance fluids (normal saline at 125 cc/hour) and empiric intravenous administration of ceftriaxone at a dose of 2 g every 24 hours (to cover for possible aspiration pneumonia) was initiated. He was transferred to our intensive care unit for further management. A nasopharyngolaryngoscopy revealed bilateral true vocal cord paralysis. After stabilization on a ventilator, he underwent a tracheostomy on day 3 of admission and was transitioned successfully to a tracheostomy collar. Following extubation, he had consistent and gradual recovery of mental status. This was not, however, accompanied by any progressive improvement in motor function. He continued to have vocal cord immobility and flaccid weakness of his extremities.

### Investigations

**Fig. 1:**
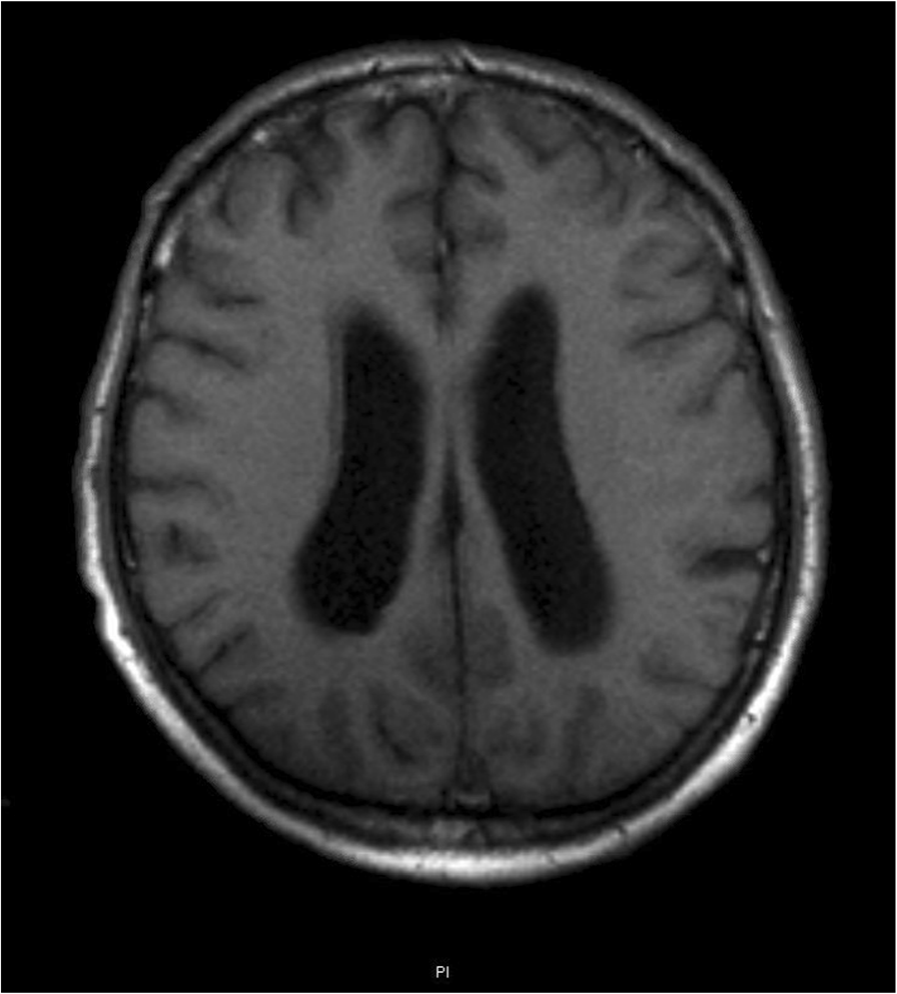
Axial T1 image of MRI Brain showing mild ventriculomegaly

On presentation, laboratory testing revealed a white blood cell (WBC) count, 13,800 cells/μL; hemoglobin, 14.7 g/dl; platelet count, 235,000 cells/μL; sodium, of 143 mmol/L; potassium, 4.2 mmol/L; chloride, 105 mmol/L; glucose, 176 mg/dL; bicarbonate, 27 mmol/L; creatinine, 0.53 mg/dL; blood urea nitrogen (BUN), 15 mg/dL; and calcium, 8.0 mg/dL. A hepatic function panel revealed a total protein level, 5.2 g/dL; serum albumin, 3.5 g/dL; total bilirubin, 0.5 mg/dl; aspartate aminotransferase (AST), 18 U/L; alanine aminotransferase (ALT), 21 U/L; international normalized ratio (INR), 1.08; creatine kinase, 127 U/L; and troponin I less than 0.015 ng/mL. Urine analysis demonstrated cloudy urine with specific gravity 1.018, pH 4.8, with 116 red blood cells (RBCs) and 4 white blood cells (WBCs), negative leukocyte esterase and nitrite. An electrocardiogram and chest X-ray were unremarkable. A urine drug screen and serum alcohol levels were negative. Serum thyroid-stimulating hormone (TSH) and cortisol levels were within normal limits. Thiamine, vitamin B6, and B12 levels were normal. Serological tests were negative for Lyme disease, human immunodeficiency virus (HIV), syphilis, hepatitis B, and hepatitis C. Blood and urine cultures obtained at presentation showed no growth. Non-contrast computed tomography (CT) of his head and magnetic resonance imaging (MRI) of his brain were normal except for mild ventriculomegaly (Fig. 1). CT angiography of his head and neck revealed patent intracranial vasculature. A repetitive nerve stimulation electromyographic (EMG) study demonstrated patchy, moderate to severe, peripheral motor nerve denervation consistent with AIDP. A lumbar puncture was performed and analysis of the obtained cerebrospinal fluid (CSF) showed albuminocytologic dissociation with less than 3 nucleated cells per mL of fluid, elevated CSF protein of 62 mg/dl, and normal glucose levels (88 mg/dl). CSF cultures did not demonstrate any growth. Anti-GQ1B antibody and anti-acetylcholine receptor (AChR) antibody levels were negative.

### Differential diagnosis

A subacute presentation with ataxia, stridor with respiratory distress, and areflexia with weakness of bilateral upper and lower extremity offers a challenging differential (Table 1).

**Table 1:** Differential diagnosis of subacute flaccid paralysis

Vascular	Ischemic or hemorrhagic stroke
Infectious	Meningitis, progressive multifocal leukoencephalopathy, brainstem encephalitis
Traumatic	Spinal cord transection
Toxic/Metabolic	Alcoholism, hypokalemia, hypocalcemia, hypomagnesemia, botulism
Autoimmune	Guillain–Barré syndrome, Miller Fisher syndrome, transverse myelitis, myasthenia gravis, Bickerstaff’s brainstem encephalitis
Neoplastic	Primary tumor involving brainstem or metastases involving brainstem

It is prudent to first rule out an acute vascular cause such as stroke, especially involving the cerebellum and brainstem; for example, for example, an absence of any lateralizing neurological signs and disturbed consciousness would rule out stroke, especially one with brainstem involvement. Next, metabolic abnormalities due to alcoholism, vitamin deficiencies, or electrolyte deficiencies would be evident on a metabolic panel and on serum vitamin and ethyl alcohol levels. An absence of a suggestive history of exposure to *Clostridium botulinum*, such as consumption of canned or tinned foods, cosmetic use of botulinum toxin injection, or intravenous drug use, significantly lowers the possibility of botulism. An absence of fever and meningeal signs, and analysis of CSF obtained on lumbar puncture, can rule out meningitis and encephalitis. Spinal cord trauma and spinal shock in the initial stages would result in flaccid paralysis of extremities aside from obvious signs of trauma. Also, concomitant bulbar paralysis is highly unlikely. Overlapping autoimmune demyelinating syndromes such as Guillain–Barré syndrome (GBS) and Bickerstaff’s brainstem encephalitis (BBE) must be considered. GBS is more likely to have a characteristic pattern of ascending paralysis. BBE is considered to lie on the same spectrum as MFS as it may present with ataxia and ophthalmoplegia and can have positive anti-GQ1b antibodies. However, BBE is characterized more by hyperreflexia and somnolence. New-onset MG can be suspected but, in our patient, motor involvement was patchy, the anti-AChR antibody test result was negative, and EMG was not suggestive of MG.

### Treatment and outcome

Based on the above, a diagnosis of MFS involving the bulbar muscles was made and our patient was started on a 5-day course of intravenous immunoglobulin at a daily dose of 0.4 mg/kg body weight per day. Following completion of this course, he showed clinical improvement in phonation, overall muscle strength, as well as tone. Although he initially did require overnight ventilatory support through his tracheostomy, the need for this gradually diminished and he was weaned off the ventilator altogether.

Subsequent follow-up laryngoscopies after 1, 2, and 4 weeks showed improvement in vocal cord abduction. His overall respiratory status also improved with decreasing oxygen requirement and decreased need for tracheostomy suctioning. He continued to have moderate pharyngeal dysphagia and required placement of a jejunostomy tube for feeding. His hospitalization was also complicated with development of aspiration pneumonia. He was eventually discharged to a skilled nursing facility on a tracheostomy collar and jejunostomy tube after a total of 6 weeks of hospitalization and subsequently discharged home after making suitable progress in physical rehabilitation. He continued to follow-up with the physical medicine and rehabilitation (PMR) out-patient clinic and remained tracheostomy-dependent at 6 months following hospitalization. He was able to ambulate with a walker at that time.

## Discussion

Apart from the classic triad of ophthalmoplegia, ataxia, and areflexia, the MFS variant of AIDP can present with uncommon symptoms such as bulbar, facial, and pupillary muscle palsies. Our patient presented with stridor and respiratory distress in addition to ataxia, areflexia, and descending motor weakness. Albuminocytologic dissociation noted on CSF analysis, unremarkable central nervous system imaging, and EMG findings suggestive of patchy peripheral nerve denervation in combination with the above clinical presentation helped to narrow the otherwise wide differential diagnosis to MFS. The case that we have summarized above is an unusual case of MFS resulting in vocal cord palsy and respiratory failure requiring mechanical ventilatory support and which improved with intravenous immunoglobulin therapy.

MFS accounts for 1–5% of AIDP cases in the West but approximately one-fifth of AIDP cases in Japan and Taiwan [2, 3]. It is associated with antibodies against GQ1b (a ganglioside component) in approximately 80–90% of cases [4]. Anti-GQ1b antibodies develop following an infection (*Campylobacter jejuni, Haemophilus influenzae*) through a process of molecular mimicry as the body mounts an immune response to a biochemically similar epitope on the bacterium [5]. The presence of this antibody shows a strong association with involvement of the third, fourth, and sixth cranial nerves. Anti-GQ1b antibody can also be present in other variants of AIDP in which there is ataxia or ophthalmoplegia at presentation [6]. Multiple cases of anti-GQ1b antibody-negative MFS have been reported in the literature [7–9]. A study by Kimoto et al. [10] demonstrated that almost 10% of MFS cases were anti-GQ1b antibody-negative. The authors suggested that antibodies against gangliosides other than GQ1b can also play a pathogenic role in the development of MFS. This could possibly explain the absence of anti-GQ1b antibodies in the case we have described.

A single-center retrospective study of 157 patients with AIDP in Taiwan demonstrated bulbar dysfunction in 19 patients with MFS but no incidence of respiratory failure [3]. Although cases of vocal cord paralysis associated with classic AIDP have been reported [11, 12], we found only one reported case of vocal cord paralysis due to MFS [13]. In that case, laryngeal muscle re-innervation and pacemaker therapy was attempted successfully to treat persistent vocal cord paralysis; however, there is no discussion of the patient receiving any medical therapy such as intravenous immunoglobulin or plasmapheresis. Among the AIDP variants, MFS is considered to have a good prognosis and is usually self-limiting [14]. A Cochrane review of MFS treatment trials demonstrated that intravenous immunoglobulin did hasten recovery, affording an almost 90% chance of complete clinical recovery at 6 months as compared to a variable recovery between 60 and 100% in patients receiving only supportive therapy [15]. It has been proposed that intravenous immunoglobulin acts by blocking the Fc receptors on macrophages, thereby preventing antibody-mediated attack on Schwann cell membranes. In addition, there is inhibition of cytokines by anti-cytokine antibodies present in the pooled sera and suppression of the complement cascade [16]. There was no evidence that corticosteroids affected the overall clinical course, but early plasmapheresis (within 4 weeks of onset of symptoms) was noted to contribute to faster recovery in both ambulant and non-ambulant patients [17]. The results from trials assessing the use of a combination of above treatments are currently awaited.

## Conclusion

An acute presentation of flaccid paralysis is always concerning and should prompt the consideration of a wide differential. Etiologies that are more common, potentially fatal, and requiring rapid treatment must be addressed first. These include stroke (as a result of brainstem ischemia), electrolyte abnormalities, and botulism. If there is persistent weakness, a wider differential that includes AIDP must be considered. There should be a high suspicion for MFS if the presentation involves ataxia, areflexia, and ophthalmoplegia with involvement of other bulbar muscles even if anti-ganglioside antibodies are not detected. Early treatment with intravenous immunoglobulin can hasten recovery and improve clinical outcomes.

## Data Availability

Authors are ready to provide all relevant data and supporting material.
